# Radiologic complete response (rCR) in contrast-enhanced magnetic resonance imaging (CE-MRI) after neoadjuvant chemotherapy for early breast cancer predicts recurrence-free survival but not pathologic complete response (pCR)

**DOI:** 10.1186/s13058-018-1091-y

**Published:** 2019-01-31

**Authors:** Simon Peter Gampenrieder, Andreas Peer, Christian Weismann, Matthias Meissnitzer, Gabriel Rinnerthaler, Johanna Webhofer, Theresa Westphal, Marina Riedmann, Thomas Meissnitzer, Heike Egger, Frederike Klaassen Federspiel, Roland Reitsamer, Cornelia Hauser-Kronberger, Katharina Stering, Klaus Hergan, Brigitte Mlineritsch, Richard Greil

**Affiliations:** 1 Department of Internal Medicine III with Hematology, Medical Oncology, Hemostaseology, Infectiology and Rheumatology, Oncologic Center; Salzburg Cancer Research Institute - Laboratory for Immunological and Molecular Cancer Research (SCRI-LIMCR), Paracelsus Medical University, Salzburg, Austria; 2 Cancer Cluster Salzburg, Salzburg, Austria; 3 Department of Radiology, Paracelsus Medical University Salzburg, Salzburg, Austria; 4 Department of Medical Statistics, Informatics and Health Economics, Medical University of Innsbruck, Innsbruck, Austria; 5 Department of Special Gynecology and Breast Center, Paracelsus Medical University Salzburg, Salzburg, Austria; 6 Department of Pathology, Paracelsus Medical University Salzburg, Salzburg, Austria

**Keywords:** Breast cancer, MRI, Neoadjuvant chemotherapy, Prediction of complete pathologic response, Survival

## Abstract

**Background:**

Patients with early breast cancer (EBC) achieving pathologic complete response (pCR) after neoadjuvant chemotherapy (NACT) have a favorable prognosis. Breast surgery might be avoided in patients in whom the presence of residual tumor can be ruled out with high confidence. Here, we investigated the diagnostic accuracy of contrast-enhanced MRI (CE-MRI) in predicting pCR and long-term outcome after NACT.

**Methods:**

Patients with EBC, including patients with locally advanced disease, who had undergone CE-MRI after NACT, were retrospectively analyzed (*n* = 246). Three radiologists, blinded to clinicopathologic data, reevaluated all MRI scans regarding to the absence (radiologic complete remission; rCR) or presence (no-rCR) of residual contrast enhancement. Clinical and pathologic responses were compared categorically using Cohen’s kappa statistic. The Kaplan-Meier method was used to estimate recurrence-free survival (RFS) and overall survival (OS).

**Results:**

Overall rCR and pCR (no invasive tumor in the breast and axilla (ypT0/is N0)) rates were 45% (111/246) and 29% (71/246), respectively. Only 48% (53/111; 95% CI 38–57%) of rCR corresponded to a pCR (= positive predictive value - PPV). Conversely, in 87% (117/135; 95% CI 79–92%) of patients, residual tumor observed on MRI was pathologically confirmed (= negative predictive value - NPV). Sensitivity to detect a pCR was 75% (53/71; 95% CI 63–84%), while specificity to detect residual tumor and accuracy were 67% (117/175; 95% CI 59–74%) and 69% (170/246; 95% CI 63–75%), respectively. The PPV was significantly lower in hormone-receptor (HR)-positive compared to HR-negative tumors (17/52 = 33% vs. 36/59 = 61%; *P* = 0.004). The concordance between rCR and pCR was low (Cohen’s kappa − 0.1), however in multivariate analysis both assessments were significantly associated with RFS (rCR *P* = 0.037; pCR *P* = 0.033) and OS (rCR P = 0.033; pCR *P* = 0.043).

**Conclusion:**

Preoperative CE-MRI did not accurately predict pCR after NACT for EBC, especially not in HR-positive tumors. However, rCR was strongly associated with favorable RFS and OS.

**Electronic supplementary material:**

The online version of this article (10.1186/s13058-018-1091-y) contains supplementary material, which is available to authorized users.

## Condensed abstract

Radiologic complete response (rCR) on contrast-enhanced MRI (CE-MRI) after neoadjuvant chemotherapy did not accurately predict pathologic complete response (pCR) in early breast cancer. Nevertheless, rCR was strongly associated with recurrence-free and overall survival and added prognostic information to the pathologic risk classification.

## Introduction

Neoadjuvant chemotherapy (NACT) has become standard care for most patients with high-risk early breast cancer (EBC). This is especially true for triple-negative and human epidermal growth factor receptor (HER2)-positive disease, where high rates of pathologic complete response (pCR) can be achieved [1–3]. Multiple trials have shown that pCR in the breast and axilla is associated with a favorable prognosis independent of the breast cancer subtype [3]. However, patients undergoing NACT have been shown to have a moderately higher risk of local recurrence after 15 years compared to patients treated with adjuvant chemotherapy (21.4% vs. 15.9%; rate ratio 1.37; 95% CI 1.17–1.61; *P* = 0.0001) [4]. This might - at least in part - be attributed to the higher rate of breast conservation after NACT [4], where potentially residual disease is not resected. As the removal of the radiological residual area of the primary tumor is considered standard care after NACT [5], it is important that preoperative imaging detects residual cancer with high precision in order to guarantee adequate surgery.

Magnetic resonance imaging (MRI) has been shown to be the most accurate diagnostic tool for breast cancer diagnosis and follow-up care [6] and provides greater accuracy for tumor size prediction than mammography and ultrasound in patients not receiving neoadjuvant therapy [7]. Several studies have investigated the diagnostic power of breast MRI during and/or after NACT [8–13]. Most of these trials and the meta-analysis of Marinovich et al. [12] demonstrated high sensitivity for MRI to correctly detect residual tumor after NACT, but specificity was rather low (correct identification of pCR). In the future, breast surgery might be avoided in patients in whom the presence of residual tumor after neoadjuvant therapy can be ruled out with very high confidence.

In this retrospective monocentric study, we investigated the diagnostic accuracy of contrast-enhanced MRI (CE-MRI) to predict pCR after NACT. Unlike most of the previously reported studies, we used a blinded design and follow-up data allowed us to correlate radiologic response with recurrence-free survival (RFS) and overall survival (OS).

## Material and methods

### Patients

Patients with early breast cancer (EBC), including patients with locally advanced but operable disease, treated with NACT between September 2006 and May 2016, followed by CE-MRI and undergoing breast-conserving surgery or mastectomy at our tertiary cancer center, were included in this retrospective study. NACT was given according to the local standard based on international guidelines (Table 1). Patients with insufficient NACT, defined as less than 3 months of treatment, and patients with insufficiently low MRI quality were excluded.

**Table 1:** Patient characteristics

Characteristics		Study population (*n* = 246)	
Median age (range), years		50 (23–81)	
Stage	cT1	59	24%
	cT2	140	57%
	cT3/4	47	19%
	cN0	120	49%
	cN+	124	50%
	Nx	2	1%
Tumor grading	G1–2	106	43%
	G3	139	57%
Ki-67	≤ 20%	48	20%
	> 20%	154	63%
	Unknown	44	18%
Subtype^a^	Luminal A-like	57	23%
	Luminal B-like	29	12%
	HER2+/HR-	33	13%
	HER2+/HR+	37	15%
	Triple-negative	90	37%
Histology	Ductal	201	82%
	Lobular	17	7%
	Other subtypes	28	11%
Neoadjuvant therapy	Anthracycline-based and taxane-based	225	92%
	Anthraycycline-based	2	< 1%
	Taxane-based	18	7%
	Trastuzumab	67^b^	27%
	Pertuzumab	23^c^	9%
Type of surgery	Breast conserving	179	73%
	Mastectomy	66	27%
MRI	Baseline MRI	234	95%
	1.5 Tesla after NACT	112	45%
	3.0 Tesla after NACT	123	50%
Radiologic response	rCR	111	45%
	rPR	121	49%
	rSD	8	3%
	rPD	6	2%
Pathologic response	pCR	71	29%
	no pCR	175	71%
Adjuvant therapy	Additional chemotherapy	5	2%
	Endocrine therapy	109^d^	44%
	Trastuzumab	63^e^	26%

*NACT* neoadjuvant chemotherapy, *rCR* radiologic complete response, *rPR* radiologic partial response, *rSD* radiologic stable disease, *rPD* radiologic progressive disease, *pCR* pathologic complete response, *HER2* human epidermal growth factor receptor 2, *MRI* magnetic resonance imaging

^a^Defined by immunohistochemical analysis: luminal A-like was defined as hormone receptor (HR)-positive, HER2 negative, grade (G) 1–2 and Ki-67 < 20% (if available); luminal B-like was defined as HR-positive, HER2 negative, G2 and Ki-67 ≥ 20% or G3

^b^96% of patients with HER2-positive tumors

^c^33% of patients with HER2-positive tumors

^d^85% of patients with HR+ tumors

^e^87% of patients with HER2-positive tumors

### MRI imaging

All except 11 breast MR exams (96%) were performed on a 3-Tesla or 1.5-Tesla scanner (Philips Achieva® and Philips Ingenia® systems, Philips Healthcare, Best, The Netherlands). We used a dedicated phased-array breast coil with the patients lying prone. We obtained axial T2-weighted fat-suppressed images (echo time (TE)/repetition time (TR)/inversion recovery (IR) of 60/9065/230 msec; with a slice thickness of 3 mm and a field of view of 30–40 cm) and axial diffusion-weighted images (TE IR/TR of 59/8157 msec; b-values up to 600; slice thickness 3 mm). Finally, four axial T1-weighted fat-suppressed dynamic acquisitions (TE/TR, 2.3/4.1 msec with a slice thickness of 1 mm) were registered over the duration of 5 min after intravenous contrast medium injection. As gadolinium contrast agent we used either 15 mL gadoteric acid (DOTAREM®, Guerbet, Cedex, France) or 7 mL gadobutrol (GADOVIST®, Bayer Schering Pharma, Berlin, Germany followed by a saline bolus flash. The remaining 11 breast MR exams were performed in external institutes using scanners of less than 1.5 Tesla.

### MRI reading

Imaging interpretation was performed by three radiologists specializing in breast imaging (CW, TM, HE), with 20, 12 and 10 years of experience in interpreting breast MRI, respectively. All readers reevaluated the preoperative MRI scans (one exam interpreted by one radiologist) blinded to clinical and pathologic data. Baseline MRI scans were used for comparison when available or alternatively, mammograms and ultrasound images were used. Radiologic complete response (rCR) was defined as absence on visual inspection of contrast enhancement on any serial image of dynamic contrast-enhanced T1-weighted MRI. Any amount of enhanced area was diagnosed as no-rCR including “partial response”, “stable disease” and “progressive disease” according to the Response Evaluation Criteria in Solid Tumors (RECIST) 1.1 guidelines. The single longest dimension of the largest residual lesion was measured for comparison with the pathology report.

### Pathology

Pathologic examinations were performed by experienced breast pathologists according to the local standard. pCR was defined as no invasive tumor in the breast and axilla (ypT0/is N0)l however three alternative definitions were investigated as well: no invasive or non-invasive tumor in breast and axilla (ypT0/N0), no invasive tumor in the breast (ypT0/is) and no invasive or non-invasive tumor in the breast (ypT0) irrespective of lymph node involvement. The single longest dimension of the largest residual tumor lesion was used for size comparison with MRI. Residual cancer burden (RCB) was assessed according to predefined standards. [14]

### Definition of diagnostic accuracy

Sensitivity (true positive (TP) rate) was defined as the probability by which a pCR can be detected by MRI (TP/(TP + false negative (FN))); specificity (true negative (TN) rate) was defined as probability by which a no-pCR can be detected by MRI (TN/(false positive (FP) + TN)); positive predictive value (PPV) was defined as probability by which rCR predicts pCR (TP/(TP + FP)) and negative predictive value (NPV) was defined as probability by which no-rCR predicts no-pCR (TN/(FN + TN)). Accuracy (ACC) was defined as proportion of true results among the total number of cases examined ((TP + TN)/all) (Additional file 1: Figure S1).

### Statistical analysis

*P* values for comparison of various patient subgroups were calculated using Fisher’s exact test. Clinical and pathologic responses were compared categorically using Cohen’s kappa statistic (≥ 0.81 excellent agreement, 0.41–0.80 moderate to good agreement, ≤ 0.4 fair to poor agreement) [15]. Spearman rank correlation analysis and Cohens’ *d* were used to measure the relationship between the preoperative radiologic tumor measurement after NACT and the residual pathologic tumor size (Spearman rho: 0 no correlation, ≥ 0.75 strong correlation, 1 perfect correlation; Cohens’ *d*: 0.2 small difference, 0.5 moderate difference, 0.8 strong difference between the means) [16]. Wilcoxon signed rank test was used to assess whether the measurement mean ranks differed. The Kaplan-Meier method was used for estimates of RFS and OS and hazard ratios were calculated for subgroup comparisons. Univariate Cox regression analyses of RFS included the clinicopathologic factors pCR (yes vs. no), rCR (yes vs. no), American Joint Committee on Cancer (AJCC, version 7) stage (I + IIA vs. IIB + III), grading (1 + 2 vs. 3), Ki-67 (≤ 20% vs. > 20%), HR status (positive vs. negative), HER2 status (positive vs. negative) and menopausal status (premenopausal or perimenopausal vs. postmenopausal). All statistically significant variables from univariate analysis were included in the multivariate analysis. pCR, rCR and statistically significant clinicopathologic parameters in univariate RFS Cox regression analysis were combined. Ability of these combinations to help discriminate between patients with or without recurrence was assessed by receiver operating characteristics (ROC) analysis and corresponding *c*-indices computed from the predicted probabilities in the corresponding binomial logistic regression models in the full dataset and were validated by 10-fold cross-classification. *P* values reflecting the differences between the prediction performance of binomial logistic regression models were calculated using the Wilcoxon test. No adjustment for multiple testing was performed, because of the exploratory character of the study.

### Endpoints

The co-primary endpoints of the analysis were PPV and NPV of the preoperative MRI for prediction of pCR and no-pCR, respectively, in the overall cohort. All survival endpoints were defined according to the STEEP system [17]. Patients alive (for OS) and who had not experienced recurrence (for RFS) at the data cutoff date were censored at the last follow-up date.

## Results

**Fig. 1:**
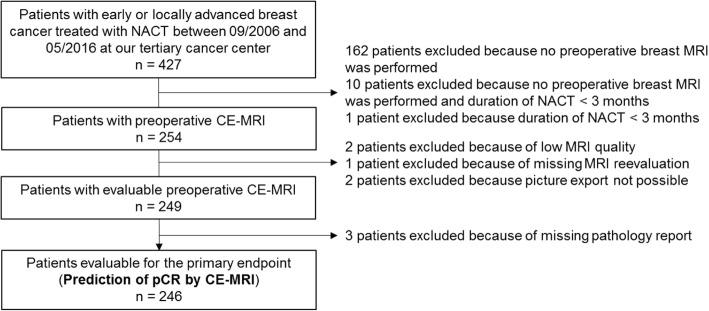
Flow diagram. NACT, neoadjuvant chemotherapy; MRI, magnetic resonance imaging; CE-MRI, contrast-enhanced magnetic resonance imaging; pCR, pathologic complete response

Overall, 246 patients treated with adequate NACT who had an evaluable preoperative CE-MRI scan were included in this analysis (Fig. 1). All included patients were women. The median time between CE-MRI and surgery was 14 days (range 1–142 days). Overall, rCR and pCR rates were 45% (111/246) and 29% (71/246), respectively. The highest rCR and pCR rates were observed in the triple-negative (41/90 = 46% and 33/90 = 37%) and HR-/HER2+ subgroup (18/33 = 60% and 16/33 = 49%, respectively). The lowest pCR rate was seen in HR+/HER2- tumors (11/86 = 13%), while the rCR rate was much higher in this subgroup (32/86 = 37%).

### Prediction of pCR by CE-MRI

**Fig. 2:**
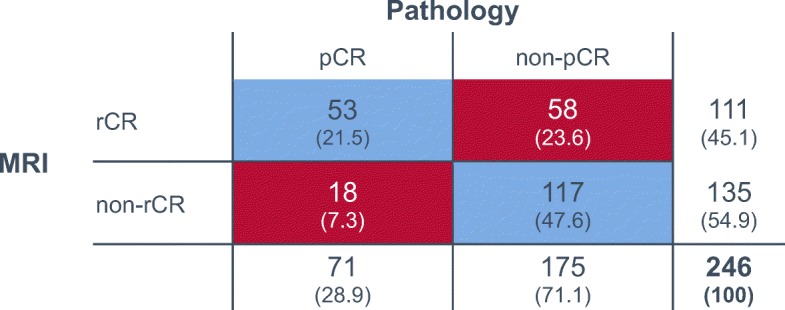
Cross-tabulation with numbers and percentages (in brackets) for radiologic complete response (rCR)/no rCR and pathologic complete response (pCR)/no pCR for the whole cohort

**Fig. 3:**
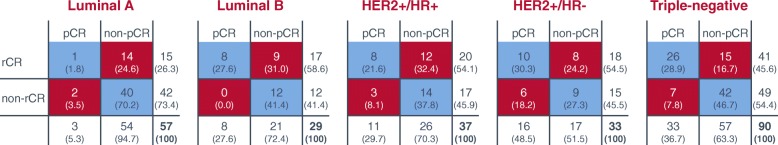
Cross-tabulation with numbers and percentages (in brackets) for radiologic complete response (rCR)/no rCR and pathologic complete response (pCR)/no pCR for breast cancer subtypes

The concordance between the radiologic and pathologic response classifications was low (Cohen’s kappa − 0.1). Only 48% (53/111; 95% CI 38–57%) of rCR corresponded to a pCR (= PPV; Table 2 and Fig. 2). Conversely, in 87% (117/135; 95% CI 79–92%) of patients, residual tumor in the MRI was pathologically confirmed (= NPV). The sensitivity to detect a pCR was 75% (53/71; 95% CI 63–84%), while specificity and accuracy were 67% (117/175; 95% CI 59–74%) and 69% (170/246; 95% CI 63–75%), respectively (Table 2). The diagnostic performance of CE-MRI to predict treatment response varied between different histologic and molecular tumor subtypes (Tables 2 and 3 and Fig. 3). The PPV was significantly lower in the HR-positive subgroup compared with the HR-negative subgroup (17/52 = 33% vs. 36/59 = 61%; *P* = 0.004) and was especially low in luminal-A-like (7%) and lobular carcinomas (0%) (Table 3). The NPV, in contrast, was significantly higher in the HR-positive subgroup (93% vs. 80%; *P* = 0.04). Interestingly, there was little difference between the four different pCR definitions (Table 4). The diagnostic performance did not vary significantly between 3.0-Tesla and ≤ 1.5-Tesla scanners (PPV 46% vs. 49%, *P* = 0.848). Furthermore, the NPV remained stable over time, while there were numerical differences in PPV at different time intervals; however, no trend towards improvement over time was evident (Additional file 1: Figure S2).

**Table 2:** Diagnostic performance of preoperative CE-MRI in different molecular tumor subtypes

Percentage (95% CI)	All	Luminal A	Luminal B	HER2+/HR+	HER2+/HR-	TNBC	*P* value^*^
pCR rate	29%	5%	28%	30%	49%	37%	
Sensitivity	75% (63–84%)	33% (1–91%)	100% (56–100%)	73% (39–94%)	63% (35–85%)	79% (61–91%)	0.120
Specificity	67% (59–74%)	74% (60–85%)	57% (32–76%)	54% (33–73%)	53% (28–77%)	74% (60–84%)	0.139
PPV	48% (38–57%)	7% (0.2–32)	47% (22–69%)	40% (19–64%)	56% (31–78%)	63% (47–78%)	0.003
NPV	87% (79–92%)	95% (84–99%)	100% (68–100%)	82% (57–96%)	60% (32–84%)	86% (73–94%)	0.009
Accuracy	69% (63–75%)	72% (58–83%)	69% (49–84%)	59% (42–75%)	58% (39–74%)	76% (65–84%)	< 0.001

*CE-MRI* contrast-enhanced magnetic resonance imaging, *pCR* pathologic complete response, *PPV* positive predictive value, *NPV* negative predictive value, *HER2* human epidermal growth factor receptor 2, *TNBC* triple-negative breast cancer

*Fisher’s exact test for all subgroups

**Table 3:** Diagnostic performance of preoperative CE-MRI in different histologic and biologic tumor subtypes

Percentage (95% CI)	Ductal	Lobular	Others	*P* value*	HR+	HR-	*P* value*
pCR rate:	33%	12%	10%		18%	40%	
Sensitivity	76% (64–86%)	0% (0–76%)	100% (11–100%)	0.120	77% (55–92%)	73% (59–85%)	0.777
Specificity	70% (62–78%)	67% (35–85%)	58% (32–77%)	0.488	65% (55–76%)	69% (57–79%)	0.631
PPV	56% (45–66%)	0% (0–47%)	20% (2–51%)	0.003	33% (20–47%)	61% (47–73%)	0.004
NPV	85% (77–91%)	83% (46–95%)	100% (65–100%)	0.536	93% (84–97%)	80% (68–89%)	0.040
Accuracy	72% (65–78%)	59% (33–81%)	62% (37–81%)	0.024	67% (58–75%)	71% (62–78%)	0.004

*CE-MRI* contrast-enhanced magnetic resonance imaging, *pCR* pathologic complete response, *PPV* positive predictive value, *NPV* negative predictive value

*Fisher’s exact test for all subgroups

**Table 4:** Diagnostic performance of preoperative CE-MRI when different pCR definitions are used

Percentage (95% CI)	ypT0/is N0	ypT0/is	ypT0 N0	ypT0	*P* value
pCR rate:	29%	32%	24%	28%	
Sensitivity	75% (63–84%)	73% (62–82%)	76% (63–86%)	75% (63–85%)	0.980
Specificity	67% (59–74%)	68% (60–75%)	65% (57–72%)	66% (59–73%)	0.938
PPV	48% (38–57%)	51% (42–61%)	41% (31–50%)	46% (36–57%)	0.443
NPV	87% (80–92%)	84% (77–90%)	90% (83–94%)	87% (81–92%)	0.666
Accuracy	69% (63–75%)	70% (63–75%)	67% (61–73%)	69% (62–74%)	0.660

*CE-MRI* contrast-enhanced magnetic resonance imaging, *pCR* pathologic complete response, *PPV* positive predictive value, *NPV* negative predictive value, *ypT0/is N0* no invasive tumor in breast and axilla, *ypT0/is* no invasive tumor in the breast, *ypT0 N0* no invasive or non-invasive tumor in the breast and axilla, *ypT0no* invasive or non-invasive tumor in the breast

*Fisher’s exact test for all subgroups

### False positive rCR

In the case of false positive rCR (rCR but no pCR; *n* = 58), median pathologic tumor size was 0.7 cm (range 0.1–5.0 cm), residual tumor size was ≤ 1.0 cm in 66% of patients, median residual cancer burden (RCB) class was I (range I–III) and median RCB score was 1.27 (Additional file 1: Figure S3). False positive cases in CE-MRI were seen across all breast cancer subtypes with a similar distribution (23/63 = 27% luminal-like, 12/25 = 32% HR+/HER2+, 8/25 = 24% HR-/HER2+, 15/75 = 17% triple-negative; *P* = 0.21). False positive rCR did not lead to a statistically significantly higher rate of follow-up resections during the initial breast conserving operation (49.0% vs. 44.5%; *P* = 0.621) or to a higher rate of second surgery because of positive resection margins (6.9% vs. 10.1%; *P* = 0.609). The mastectomy rate was even lower in patients with rCR but no pCR (33.0% vs. 15.5%; *P* = 0.012).

### Prediction of tumor size by MRI

We found a moderate relationship between tumor size in CE-MRI and pathology reports when there was neither rCR nor pCR (*n* = 117; Spearman Rho 0.57; *P* < 0.001, Additional file 1: Figure S4). MRI accurately predicted the pathologically assessed longest tumor diameter to within 1 cm in 67% of patients, underestimated tumor size by more than 1 cm in 8% of patients and overestimated tumor size by more than 1 cm in 26% of patients. This resulted in a small to moderate mean difference of 0.76 between the pathology report and MRI measurement according to Cohen [16] (Cohen’s d = 0.36 (95% CI 0.13–0.73)). The Wilcoxon signed rank test showed a statistically significant difference (*P* = 0.001).

### Prediction of long-term outcome by pCR and MRI response

**Fig. 4:**
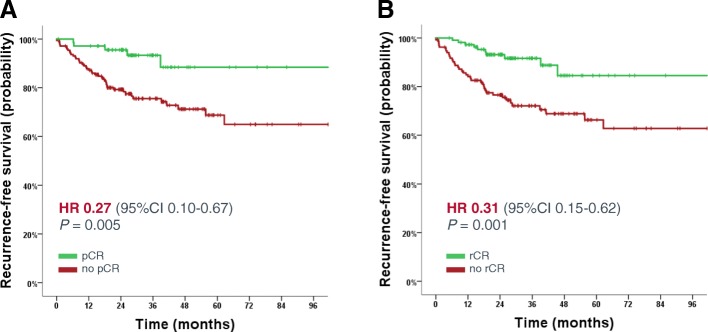
Kaplan-Meier curves for recurrence-free survival according to pathologic complete response (pCR) (**a**) and radiologic complete response (rCR) (**b**) after neoadjuvant chemotherapy

**Fig. 5:**
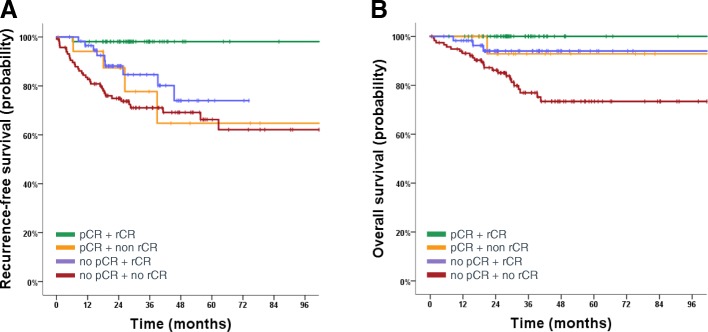
Kaplan-Meier curves for recurrence-free survival (RFS) (**a**) and oveall survival (OS) (**b**) according to pathologic complete response (pCR) and radiologic complete response (rCR), respectively. Patients achieving both pCR and rCR (*n* = 53) had an excellent prognosis for both RFS (1 event) and overall survival (0 events). Patients achieving a pCR but no rCR (*n* = 18) had a significantly higher risk of recurrence (4 events), but not of death (1 event)

After a median follow-up of 31 months (95% CI 28.2–36.2 months) there were 47 recurrences (33 distant, 5 local and 9 both) and 28 deaths. In the overall cohort, RFS and OS rates at 3 years were 82.9% and 89.4%, respectively. Patients achieving a pCR had a significantly lower risk of recurrence or death (3-year RFS 94.4% vs.78.3%, HR 0.27; 95% CI 0.10–0.67; *P* = 0.005; Fig. 4). The same effect was observed for patients with rCR, with a hazard ratio in the same range, indicating strong prognostic value (3-year RFS with rCR 92.8% vs. 74.8% without rCR, HR 0.31; 95% CI 0.15–0.62; *P* = 0.001; Fig. 4). Patients with both rCR and pCR (*n* = 53) had an excellent prognosis irrespective of the tumor subtype (one event for RFS and 0 events for OS; Fig. 5). Interestingly, patients with pCR but no rCR had a significantly higher risk of recurrence (HR 12.3, 95% CI 1.37–110.7; *P* = 0.025), however, the patient number was small in this subgroup (*n* = 18), which limits any firm conclusion.

In multivariate Cox regression analyses, both pCR and rCR were significantly associated with RFS (*P* = 0.033 and *P* = 0.037, respectively; Table 5). To assess the prognostic performance and individual contribution to the prediction of recurrence, c-indices were calculated from ROC analyses for pCR, for the clinicopathologic parameters (CPP) significant in univariate analysis, for rCR and combinations of these variables (Additional file 1: Figure S5). pCR had a c-index of 0.615. The addition of the CCP resulted in a c-index of 0.746 and statistically significantly increased the prognostic performance (*P* < 0.001). The prognostic performance was slightly, but not significantly, improved when the information on rCR was added to both models (c-index 0.681 (*P* = 0.298) and 0.768 (*P* = 0.133); Additional file 1: Figure S5). As expected, c-indices estimated by 10-fold cross-classification were somewhat lower than the values that were estimated in the full data set (Additional file 1: Figure S5). The validated c-indices present stable estimates in order to generalize the results of the ROC analysis.

**Table 5:** Univariate and multivariate analysis of RFS and OS including the most important clinicopathologic parameters

Variable	RFS			Multivariate			OS			Multivariate		
	Univariate						Univariate					
	*P* value*	HR	95% CI	*P* value*	HR	95% CI	*P* value*	HR	95% CI	*P* value*	HR	95% CI
pCR	**0.003**	3.77	1.49–9.51	**0.033**	2.91	1.09–7.78	**0.003**	11.32	1.54–88.34	**0.043**	8.29	1.07–64.00
rCR	**< 0.001**	3.26	1.62–6.55	**0.037**	2.17	1.05–4.48	**< 0.001**	6.70	2.02–22.20	**0.033**	3.78	1.11–12.87
AJCC stage	**0.001**	0.33	0.17–0.64	**0.004**	0.37	0.19–0.73	**0.016**	0.35	0.15–0.82	**0.076**	0.45	0.19–1.09
Grading	0.635	0.87	0.49–1.55				0.432	0.74	0.35–1.58			
Ki-67	0.909	1.05	0.47–2.32				0.366	1.75	0.51–6.03			
HR status	**0.029**	1.90	1.06–3.41	**< 0.001**	2.99	1.64–5.47	**0.003**	3.33	1.41–7.83	**< 0.001**	5.33	2.24–12.66
HER2 status	0.652	1.16	0.61–2.19				0.073	2.55	0.88–7.34			
Menopausal status	0.964	0.99	0.54–1.81				0.351	1.44	0.67–3.13			

*pCR* pathologic complete response, *rCR* radiologic complete response, *AJJC* American Joint Committee on Cancer, *RFS* recurrence-free survival, *OS* overall survival, *HR* hormone receptor, *HER2* human epidermal growth factor receptor 2

*Log rank test (Mantel-Cox)

In different breast cancer subtypes the association between pCR and long-term outcomes was strong in patients with triple-negative breast cancer (*n* = 90; HR 0.06, 95% CI 0.01–0.46; *P* < 0.007), while in the HR+/HER2- subgroup (*n* = 86; HR 0.04, 95% CI 0.00–194.71; *P* = 0.460) and in the HER2+ subgroup, no significant association between pCR and RFS was detected (*n* = 70; HR 0.66, 95% CI 0.20–2.15; *P* = 0.492; Additional file 1: Figure S6). Regarding MRI, Regarding MRI, there was a strongly significant association between rCR and RFS in patients with triple-negative breast cancer as well (HR 0.15, 95% CI 0.05–0.51; *P* < 0.001). In the HR+/HER2- subgroup the results was borderline significant (HR 0.15, 95% CI 0.02–1.19; *P* = 0.073). Similar to pCR, there was no association between rCR and outcome in the HER2+ subgroup (HR 0.74, 95% CI 0.25–2.20; *P* = 0.588; Additional file 1: Figure S6).

**Fig. 6:**
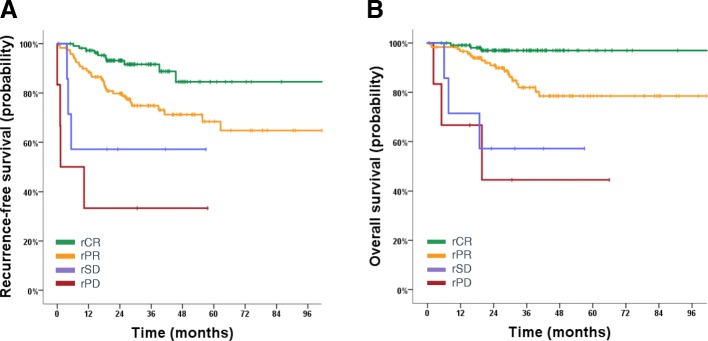
Kaplan-Meier curves for recurrence-free survival (**a**) and overall survival (**b**) according to radiologic response. rCR, radiologic complete response; rPR, radiologic partial response; rSD, radiologic stable disease; rPD, radiologic progressive disease

The amount of radiologic response predicted the risk of recurrence as well: patients with partial remission after NACT had worse prognosis than patients with rCR (HR 1.68, 95% CI 1.17–2.39, *P* = 0.003), but better prognosis than patients with stable or progressive disease (HR 0.31, 95% CI 0.13–0.69, *P* = 0.003; Fig. 6), respectively.

## Discussion

To the best of our knowledge this is the largest study investigating the value of post-neoadjuvant CE-MRI for predicting pCR using a blinded design. Unlike in most of the previous trials, follow-up data were available, allowing us to test correlation between radiologic response and risk of recurrence or death. In accordance with many other studies [8, 10–13, 18, 19], the PPV for pCR prediction was by far not good enough to replace pathology examination (PPV 48%). Even in the triple-negative subgroup, where MRI performed best, the PPV was unsatisfactorily low (63%). The small tumor size identified by pathology examination in the case of false positive MRI (rCR but no pCR: median diameter 0.7 cm; median RCB score 1.27), suggests that in most of the cases, persistence of small tumor cell nests with regular vascularization were not detected on MRI.

Residual disease, in contrast, can be predicted with high accuracy by the preoperative CE-MRI (NPV 87%), especially in the luminal subtypes (NPV 95–100%). This is of importance, because surgical resection after NACT should be restricted to the radiological residual area [5]. These differences in MRI performance between different breast cancer subtypes have been reported previously in the literature and might be explained by the fact that tumor vascularization differs among subtypes. Less aggressive tumors like HR-positive or lobular carcinoma have a lower degree of angiogenesis and consequently reduced contrast enhancement on MRI compared to more aggressive breast cancer subtypes like triple-negative breast cancer [20]. Furthermore, HR-positive tumors are for example more likely non-mass-like lesions compared to triple-negative tumors and are more likely to contain lobular features [20, 21]. It is reasonable to assume that the imaging appearances on MRI also differ among breast cancers subtypes following neoadjuvant chemotherapy - an assumption supported by our data.

Several attempts have been made to improve the performance of MRI in this regard. Foremost, the addition of specific MRI sequences like diffusion-weighted imaging (DWI) have been investigated [22–24]. Furthermore, quantitative assessments of contrast enhancement intensity at the tumor bed, called lesion-to-background parenchymal signal enhancement ratio (SER), has been shown to improve differentiation between pCR and minimal residual cancer [25]. Other possibilities are the inclusion of tumor regression shrinkage patterns (concentric, nodular or mixed) [26], introduction of subtype-specific contrast enhancement thresholds [27] or integrated fluorodeoxyglucose positron emission tomography (FDG PET)/MRI for early response prediction [28].

A totally different approach to improve the predictive value for pCR is the use of fine-needle aspiration (FNA) or vacuum-assisted core biopsies (VACB) of the clip-marked area after NACT in the case of rCR. Preliminary data reveal varying results: while a prospective single-center trial at the MD Anderson showed a high PPV of 95% (95% CI 75–100%) for FNA plus VACB before surgery [29], a much lower PPV of 71–76% was reported in other trials, when restricted to patients with rCR [30, 31], Several studies are currently investigating this approach (e.g. NCT03188393, NCT02945579).

Despite the low PPV for predicting pCR, rCR was strongly prognostic with an absolute 3-year RFS benefit of 18% in patients with rCR compared to those without rCR (HR 0.31; 95% CI 0.15–0.62; *P* = 0.001). This fact could be explained by the low tumor burden in the case of false positive MRI, which probably does not affect recurrence rate and survival. Exploratory Kaplan-Meier analyses found significant survival differences between patients with rCR and patients without rCR in the triple-negative and HR+/HER2- subtypes, but not in the HER2-positive subgroup. A similar association was shown in the I-SPY 1 study [32]. In this study, radiologic tumor volume was an even stronger predictor of RFS than pCR. In contrast to our study, this effect was not seen in the triple-negative subgroup [32]. In line with our results, a Korean study, including 174 patients with breast cancer undergoing NACT, reported a similar good outcome in patients with rCR and in patients with pCR [33]. In contrast to I-SPY 1, the major effect was seen in patients with triple-negative breast cancer. A Dutch study evaluated patients with HER2-positive tumors only (*n* = 296) and found an absolute 5-year-RFS benefit of 20% in patients with rCR compared to those without rCR (HR 0.34, 95% CI 0.17–0.65; *P* = 0.001) [34]. Similar results were shown in a study including patients with HR+/HER2- disease only (*n* = 272; HR 12.81; *P* = 0.004) [35]. Since pCR is infrequently achieved in HR+/HER2- breast cancer and its association with outcome is not as strong as in other breast cancer subtypes [3], pCR is not a good primary endpoint for neoadjuvant trials in this breast cancer subtype. Given the strong prognostic value of post-treatment MRI and the higher incidence of rCR in this subtype, rCR could be a good alternative primary endpoint for future trials.

We acknowledge the following limitations. First, this was a retrospective study from a single institution. Furthermore, the follow-up period was quite short, especially for HR-positive breast cancer. This resulted in a relatively small number of recurrences, making definitive conclusions about survival outcomes difficult. Finally, we did not take into account the potential influence of further adjuvant therapy on survival. However, since 85% of patients with HR-positive tumors received adjuvant endocrine therapy, 87% of patients with HER2-positive tumors received adjuvant trastuzumab and only 5% of patients received additional adjuvant chemotherapy, this potential bias remains small. The inclusion of data obtained from MRI scanners of ≤ 1.5 Tesla is a further limitation. Since the number was small (*n* = 11) and the PPV was comparable between 3.0 Tesla and ≤ 1.5 Tesla scanners (46% vs. 49%, *P* = 0.848), the inclusion of these data is unlikely to have influenced the results essentially.

## Conclusion

Contrast-enhanced MRI does not accurately predict pCR after neoadjuvant chemotherapy in early breast cancer, especially not in HR-positive tumors. However, in the case of false rCR the dimension of residual disease is generally small. Since residual tumor tissue can be predicted with high precision, preoperative breast MRI is still of value for operation planning and could help identify patients needing longer or more intensified neoadjuvant therapy. Patients achieving a rCR generally have a good prognosis irrespective of the breast cancer subtype and the prognostic information on rCR is similar to that of pCR.

## Additional file

Additional file 1: **Figure S1.** Cross table for calculation of sensitivity, specificity, PPV, NPV and accuracy. **Figure S2.** The negative predictive value (NPV) remained stable over time, while there were numerical differences in positive predictive value (PPV) at different time intervals; however, no trend towards improvement over time was evident. **Figure S3.** Residual pathologic tumor size (a) and residual cancer burden (RCB) score (b) in false positive rCR cases (rCR but no pCR = ypT0/is): 50 of 54 cases were analyzable. The mean size of residual disease in the pathologic specimen was 0.7 cm (range 0.1–5.0 cm). The median residual cancer burden score was 1.27 (range 0.66–3.86). **Figure S4.** Correlation between MRI and pathology report for determination of tumor size. Spearman test showed strong correlation between the two reports (Spearman Rho 0.57). **Figure S5.** C-indices demonstrating the performance of pCR, cCR and different clinicopathologic parameters for recurrence prediction. The values on the x-axis are estimates of the c-index of predicted probabilities in the corresponding binomial logistic regression models. *P* values indicate whether the ability of binomial logistic regression models to discriminate between patients with and without recurrence increases significantly by addition of more variables and they were computed by means of the Wilcoxon test. Stage: AJCC anatomic stage groups, 8th edition; HR: hormone receptor status. **Figure S6.** Kaplan-Meier curves for RFS according to pathologic (pCR) (a, c, e) and radiologic complete response (rCR) (b, d, f) after NACT for three different breast cancer subtypes: HR+/HER2- (a, b), triple-negative (c, d) and HER2+ (e, f). (PDF 331 kb)

## Abbreviations

95% CI: 95% Confidence interval
AJCC: American Joint Committee on Cancer
CE-MRI: Contrast-enhanced magnetic resonance imaging
DWI: Diffusion-weighted imaging
FN: False negative
FNA: Fine-needle aspiration
FP: False positive
G: Grade
HER2: Human epidermal growth factor receptor 2
HR: Hormone receptor
MRI: Magnetic resonance imaging
NACT: Neoadjuvant chemotherapy
NPV: Negative predictive value
OS: Overall survival
pCR: Pathologic complete response
PPV: Positive predictive value
RCB: Residual cancer burden
rCR: Radiologic complete response
RECIST: Response evaluation criteria in solid tumors
RFS: Recurrence-free survival
rPD: Radiologic progressive disease
rPR: Radiologic partial remission
rSD: Radiologic stable disease
SER: Signal enhancement ratio
TN: True negative
TNBC: Triple-negative breast cancer
TP: True positive
VACB: Vacuum-assisted core biopsies
