# Supplementary material for: Radiologic complete response (rCR) in contrast-enhanced magnetic resonance imaging (CE-MRI) after neoadjuvant chemotherapy for early breast cancer predicts recurrence-free survival but not pathologic complete response (pCR)
Source: Breast Cancer Res. 2019 Jan 31;21:19. doi: 10.1186/s13058-018-1091-y (PMC6357474; doi:10.1186/s13058-018-1091-y)
Supplement: Supplementary file 1 — Figure S1. Cross table for calculation of sensitivity, specificity, PPV, NPV and accuracy. Figure S2. The negative predictive value (NPV) remained stable over time, while there were numerical differences in positive predictive value (PPV) at different time intervals; however, no trend towards improvement over time was evident. Figure S3. Residual pathologic tumor size (a) and residual cancer burden (RCB) score (b) in false positive rCR cases (rCR but no pCR = ypT0/is): 50 of 54 cases were analyzable. The mean size of residual disease in the pathologic specimen was 0.7 cm (range 0.1–5.0 cm). The median residual cancer burden score was 1.27 (range 0.66–3.86). Figure S4. Correlation between MRI and pathology report for determination of tumor size. Spearman test showed strong correlation between the two reports (Spearman Rho 0.57). Figure S5. C-indices demonstrating the performance of pCR, cCR and different clinicopathologic parameters for recurrence prediction. The values on the x-axis are estimates of the c-index of predicted probabilities in the corresponding binomial logistic regression models. P values indicate whether the ability of binomial logistic regression models to discriminate between patients with and without recurrence increases significantly by addition of more variables and they were computed by means of the Wilcoxon test. Stage: AJCC anatomic stage groups, 8th edition; HR: hormone receptor status. Figure S6. Kaplan-Meier curves for RFS according to pathologic (pCR) (a, c, e) and radiologic complete response (rCR) (b, d, f) after NACT for three different breast cancer subtypes: HR+/HER2- (a, b), triple-negative (c, d) and HER2+ (e, f). (PDF 331 kb) [file 13058_2018_1091_MOESM1_ESM.pdf]

Radiologic complete response (rCR) in contrast-enhanced MRI after neoadjuvant chemotherapy for early or locally advanced breast cancer predicts recurrence-free survival but not pathologic complete response (pCR).

Supplementary material

Figure S1

	pCR	non-pCR
rCR	True positive (TP)	False positive (FP)
non-rCR	False nevative (FN)	True negative (TN)

Figure S2

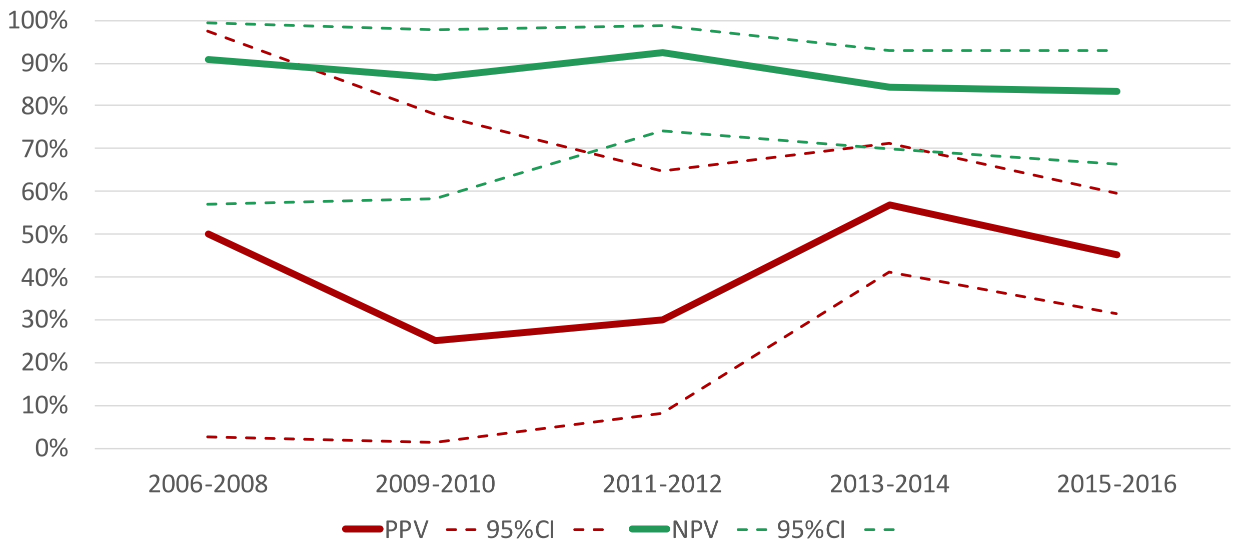

Figure S3

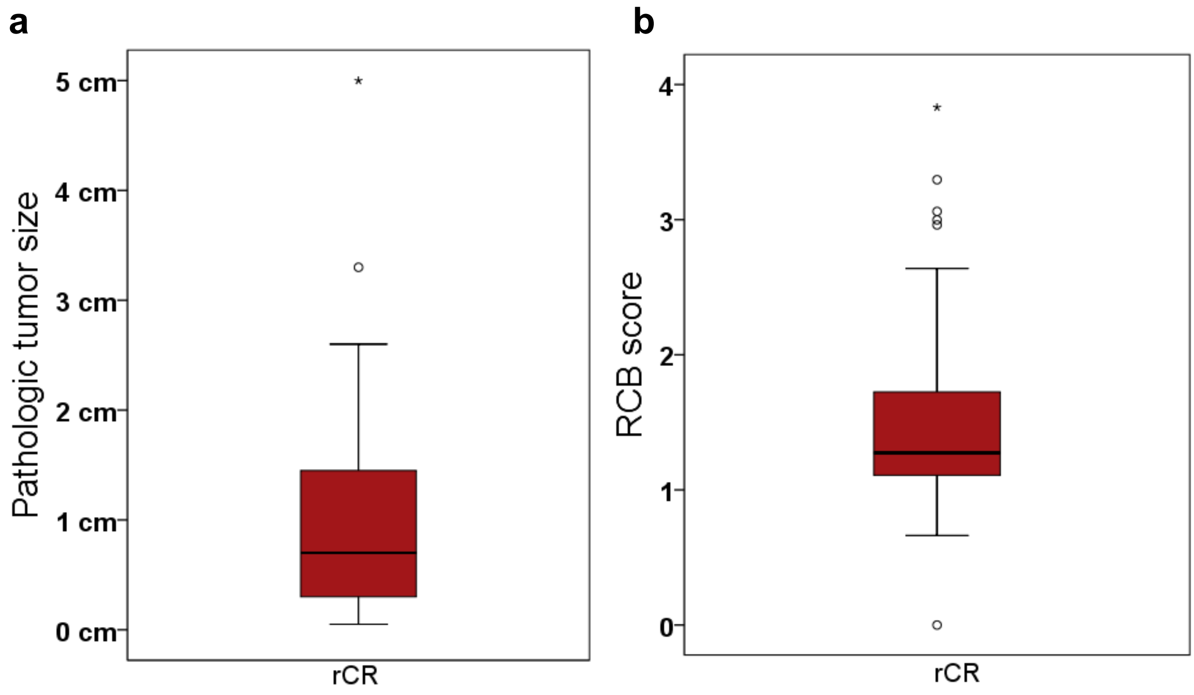

Figure S4

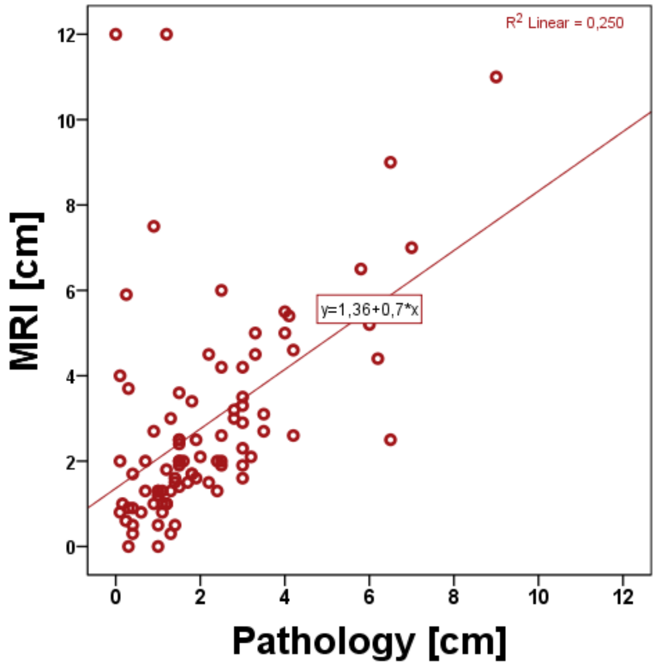

Figure S5

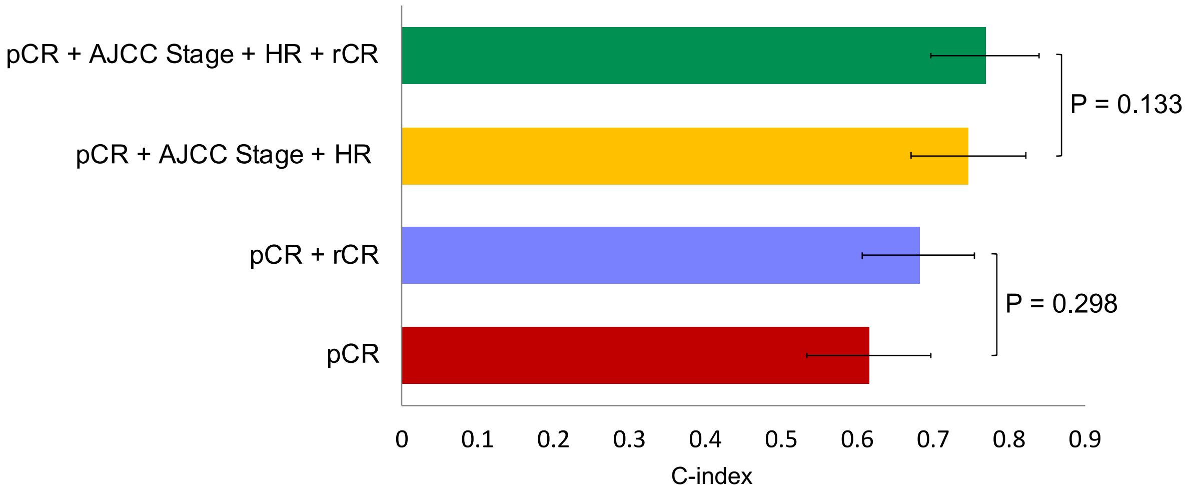

	pCR (95%CI)	pCR + rCR	pCR + AJCC + HR	pCR + AJCC + HR + rCR
c-index	0.615	0.681	0.746	0.768
95%CI	0.533-0.696	0.607-0.754	0.670-0.822	0.696-0.840
10-fold cross validation	0.586	0.639	0.699	0.749

Figure S6

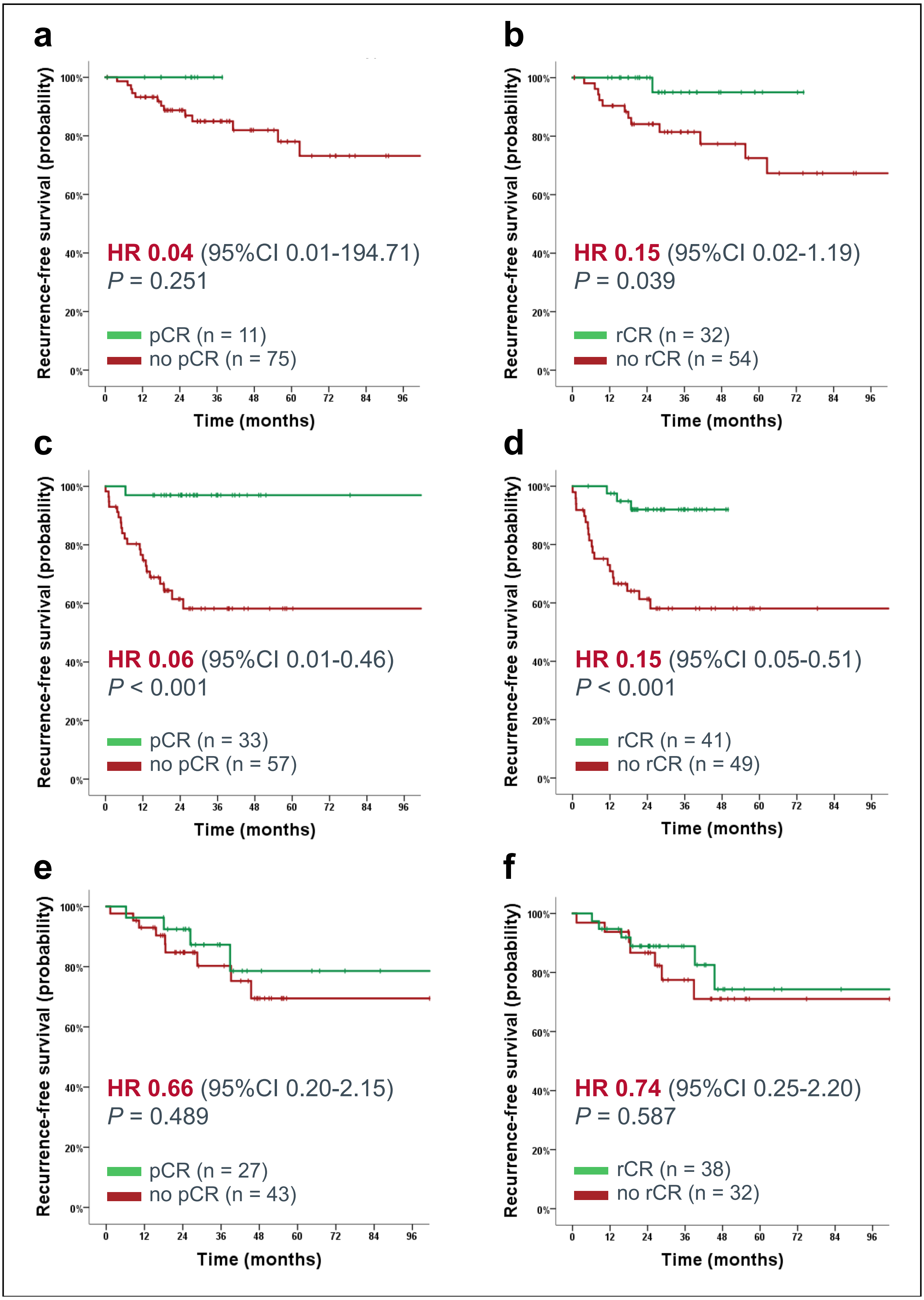
a
HR 0.04 (95%CI 0.01-194.71)
P = 0.251
pCR (n = 11)
no pCR (n = 75)
b
HR 0.15 (95%CI 0.02-1.19)
P = 0.039
rCR (n = 32)
no rCR (n = 54)
c
HR 0.06 (95%CI 0.01-0.46)
P < 0.001
pCR (n = 33)
no pCR (n = 57)
d
HR 0.15 (95%CI 0.05-0.51)
P < 0.001
rCR (n = 41)
no rCR (n = 49)
e
HR 0.66 (95%CI 0.20-2.15)
P = 0.489
pCR (n = 27)
no pCR (n = 43)
f
HR 0.74 (95%CI 0.25-2.20)
P = 0.587
rCR (n = 38)
no rCR (n = 32)
Recurrence-free survival (probability)
Time (months)
100%
80%
60%
40%
20%
0%
0
12
24
36
48
60
72
84
96
